# High temperature-induced production of unreduced pollen and its cytological effects in *Populus*

**DOI:** 10.1038/s41598-017-05661-x

**Published:** 2017-07-13

**Authors:** Jun Wang, Daili Li, Fengnan Shang, Xiangyang Kang

**Affiliations:** 1 Beijing Advanced Innovation Centre for Tree Breeding by Molecular Design, Beijing Forestry University, Beijing, 100083 People’s Republic of China; 2 National Engineering Laboratory for Tree Breeding, Beijing Forestry University, Beijing, 100083 People’s Republic of China; 3 Key Laboratory of Genetics and Breeding in Forest Trees and Ornamental Plants, MOE, Beijing Forestry University, Beijing, 100083 People’s Republic of China; 4 College of Biological Sciences and Technology, Beijing Forestry University, Beijing, 100083 People’s Republic of China; 5 Beijing Huang Fa Nursery, Beijing, 102601 People’s Republic of China

## Abstract

Temperature change is of potential to trigger the formation of unreduced gametes. In this study, we showed that short periods of high temperature treatment can induce the production of 2*n* pollen in *Populus pseudo-simonii* Kitag. The meiotic stage, duration of treatment, and temperature have significant effects on the induction of 2*n* pollen. Heat stress resulted in meiotic abnormalities, including failure of chromosome separation, chromosome stickiness, laggards and micronuclei. Spindle disorientations in the second meiotic division, such as parallel, fused, and tripolar spindles, either increased in frequency or were induced *de novo* by high temperature treatment. We found that the high temperature treatment induced depolymerisation of meiotic microtubular cytoskeleton, resulting in the failure of chromosome segregation. New microtubular cytoskeletons were able to repolymerise in some heat-treated cells after transferring them to normal conditions. However, aberrant cytokinesis occurred owing to defects of new radial microtubule systems, leading to production of monads, dyads, triads, and polyads. This suggested that depolymerisation and incomplete restoration of microtubules may be important for high temperature-induction of unreduced gametes. These findings might help us understand how polyploidisation is induced by temperature-related stress and support the potential effects of global climate change on reproductive development of plants.

## Introduction

Polyploidisation is an important driving force in plant speciation and evolution^1, 2^. It has been estimated that all angiosperms experienced at least one polyploidisation event in their lineage^3^ and allopolyploidisation and autopolyploidisation are thought to contribute to polyploid formation similarly^4^. Sexual polyploidisation, which involves unreduced (2*n*) gametes, is likely to play an important role in polyploid formation and breeding of plants^2, 5, 6^ due to heterozygosity advantage in the F_1_ generation. Based on the genetic makeup of 2*n* gametes, different cytological mechanisms of 2*n* gamete formation have been discovered, such as first division restitution (FDR), second division restitution (SDR), indeterminate meiotic restitution (IMR), and post meiotic restitution (PMR)^7–10^. These mechanisms cause different results of heterozygosity transmission from parents to progeny^11^.

The formation and frequency of 2*n* gametes could be influenced by several genetic mechanisms^2, 6, 12^. In potato, Mok and Peloquin^7^ suggested that recessive mutations controlled 2*n* pollen production by three different mechanisms: parallel spindles and premature cytokinesis 1 and 2. Mutant genes affecting meiotic synapses, the orientation of spindles, and cytokinesis were also found in potato, maize, and alfalfa, which led to production of 2*n* gametes^13^. Recently, genes that play roles in the formation of diploid or higher ploidy gametes were identified in *Arabidopsis thaliana*. d’Erfurth *et al*.^14^ found that the *AtPS1* gene controlling the formation of parallel spindles could result in the production of 2*n* pollen in *A. thaliana*. An Arabidopsis mutant *jason*, which formed parallel and fused spindles, was also found to induce FDR type 2*n* pollen production^15^.

The production of 2*n* gametes is not only governed by genetic factors, but also affected by environmental factors^16^, especially changes in temperature. Both high and low temperature stress can induce 2*n* gametes. In *Rosa*, defects in the ectexine of pollen and spindle disorientations were induced by short periods of high temperature treatment during meiosis, leading to the formation of dyads and triads^17^. Singhal *et al*.^18^ reported that the formation of 2*n* pollen in *Lindelofia longiflora* could be a consequence of the fusion of microsporocytes during the early stages of meiosis I, which was a result of low temperature stress. Mason *et al*.^19^ evaluated the interaction of temperature and genotype on 2*n* pollen production. They indicated that low temperature treatment caused significantly higher production of 2*n* pollen in *Brassica* interspecific hybrids than in the parents. In both plant and animal breeding, therefore, artificial temperature stress was usually used to induce 2*n* gametes and polyploids^20, 21^ because of its economic and nontoxic advantages.

Temperature-induced 2*n* gamete production is usually characterised by meiotic abnormalities, such as desynapsis, chromosome stickiness and missegregation, spindle disorientation, and/or cytokinesis aberrations^17, 18, 22^. The cytoskeleton plays an important role in meiotic chromosomal behaviours, nuclear division, and cytokinesis^23–27^. Temperature stress may change the location and distribution of the cytoskeleton to produce 2*n* gametes. De Storme *et al*.^28^ found that a short period of cold stress could destabilise the postmeiotic radial microtubule arrays and lead to defects in postmeiotic cytokinesis and cell wall formation, resulting in the production of 2*n* and polyploid pollen in Arabidopsis. However, the response of the cytoskeleton to heat is poorly understood.

Species and hybrids of the genus *Populus* L. are widely cultivated and utilised as an important source of fuel, fibre, and lumber in the northern hemisphere^29^. Since Nilsson-Ehle^30^ first discovered the natural triploid *Populus tremula* ^31^, characterised by extremely large leaves and fast growth, 2*n* gamete induction and utilization were concerned in breeding program of *Populus*. Heat is an important mutagenic factor for induction of 2*n* gametes of *Populus* ^32–35^. In the induced pollen, however, a high frequency of shrunken pollen grains was observed^33, 36^. Li and Zhang^36^ found that the structure of the anthers changed because of the heat shock. However, the mechanisms that lead to high temperature-induced changes in gamete development are not well known.

In this study, we analysed the effects of meiotic stage, temperature, and duration of treatment on unreduced gamete induction in *Populus pseudo-simonii* Kitag. and demonstrated high temperature-induced cytological abnormalities. Furthermore, we investigated microtubular changes as a result of high temperature treatment using indirect immunofluorescence analyses to clarify the cytological mechanisms of high temperature-induced 2*n* pollen formation.

## Results

### Response of pollen size and morphology to high temperature-treatment

**Figure 1:**
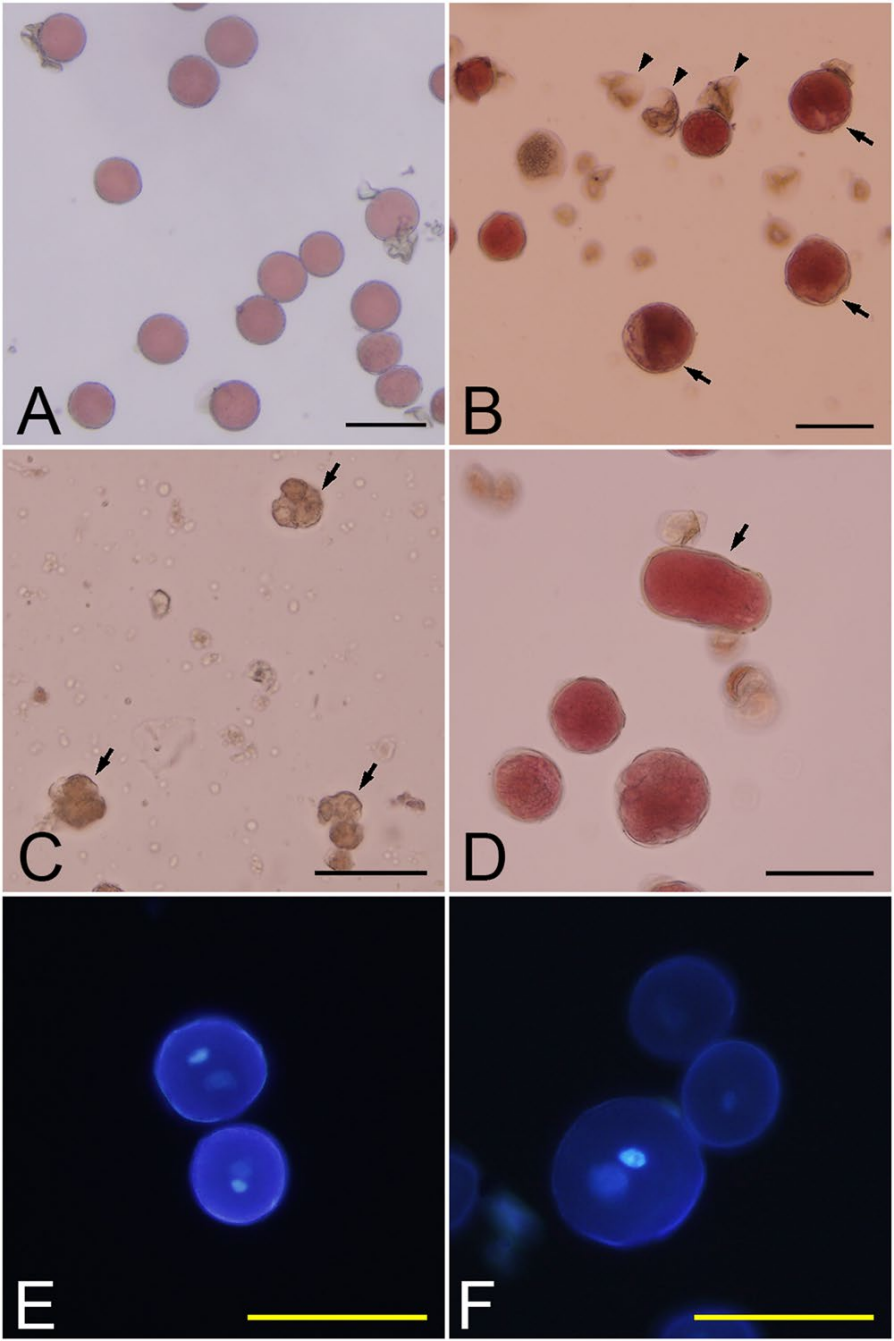
High temperature-induced pollen and untreated control pollen. (**A**) Pollen collected from untreated buds; (**B**) High temperature-induced pollen, showing large pollen grains (arrows) and aborted pollen grains (arrow heads); (**C**) Tetrad shaped aborted grains failed in cytokinesis (arrows); (**D**) Conjoined grains failed in microspore segregation (arrow); (**E**) Control pollen with DAPI staining; (**F**) High temperature-induced pollen with DAPI staining, showing a large grain with large gametophytic nuclei. Bars = 50 μm.

Compared to control pollen grains, high temperature-treated pollen grains varied greatly. In the control sample, spherical pollen grains were dominant, with 21.40% (±1.52) aborted grains (Fig. 1A). In the high temperature-induced samples, a number of large spherical grains were observed and the frequency of aborted pollen was increased (Fig. 1B). Several aborted grains maintained a tetrad shape (Fig. 1C) in the treated samples. In addition, conjoined grains (Fig. 1D) could be found in the treated pollen but was rarely found in control samples, suggesting that heat shock may have caused a failure of microspore separation.

**Figure 2:**
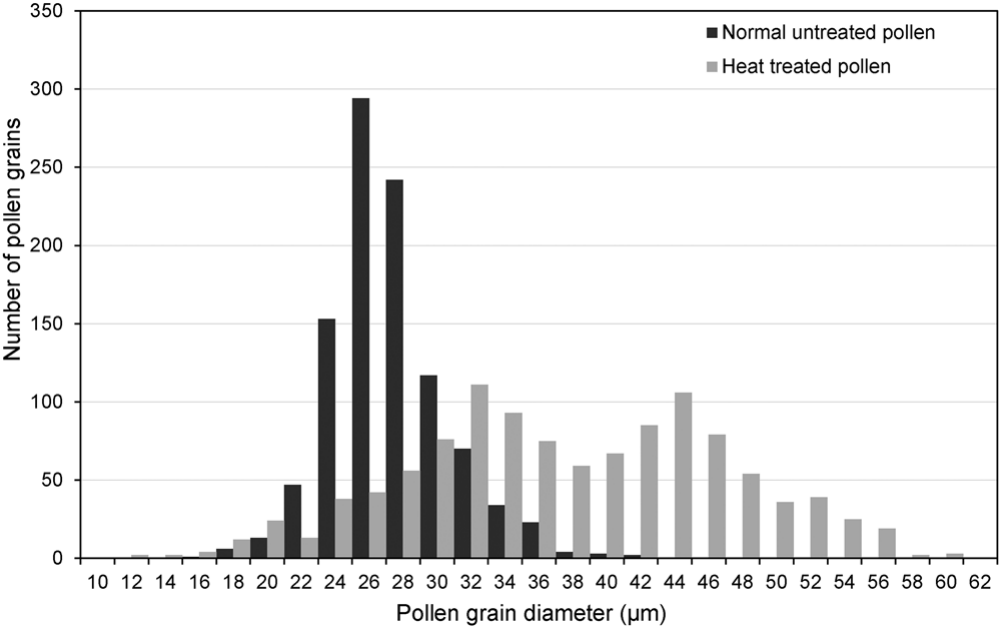
Frequency distributions of pollen grain diameters from the high temperature-treated buds and untreated control buds.

A frequency distribution histogram of pollen diameters was established based on measurements of 1009 spherical pollen grains from control samples and 1122 grains from catkins treated at 38 °C for 2 h during the diakinesis to metaphase I (DM) stage. This histogram indicates that the distribution model differed between the control and the high temperature-induced pollen samples (Fig. 2). The pollen diameter of the control, which ranged from 15.4 to 41.8 μm with an average of 27.33 ± 0.10 μm, followed an approximate Gaussian distribution. In contrast, the frequency of diameters in high temperature-induced pollen was a bimodal distribution with a range from 11.7 to 60.6 μm, which was wider than that of the control, and with a big average size at 37.64 ± 0.27 μm. There was a significant difference in pollen diameter between control and high temperature-induced samples (Wilcoxon rank test, *P* < 0.001), indicating that the high temperature treatment was able to increase pollen size. In the high temperature-induced pollen, 51.16% of grains had a diameter larger than the threshold of 37 μm, suggesting that pollen with 2*n* and higher ploidy could be induced by high temperature treatment.

Fluorescence microscopy analysis showed that both normal and large pollen grains were two-celled with one highly condensed generative nucleus and one less-condensed vegetative nucleus (Fig. 1E and F). It was clear that both types of the gametophytic nuclei in large pollen grains were significantly bigger than that of the haploid controls, suggesting that the large pollen grains were likely to be diploid or polyploid.

### Effects of meiotic stage, duration, and temperature on high temperature-induced pollen variation

**Figure 3:**
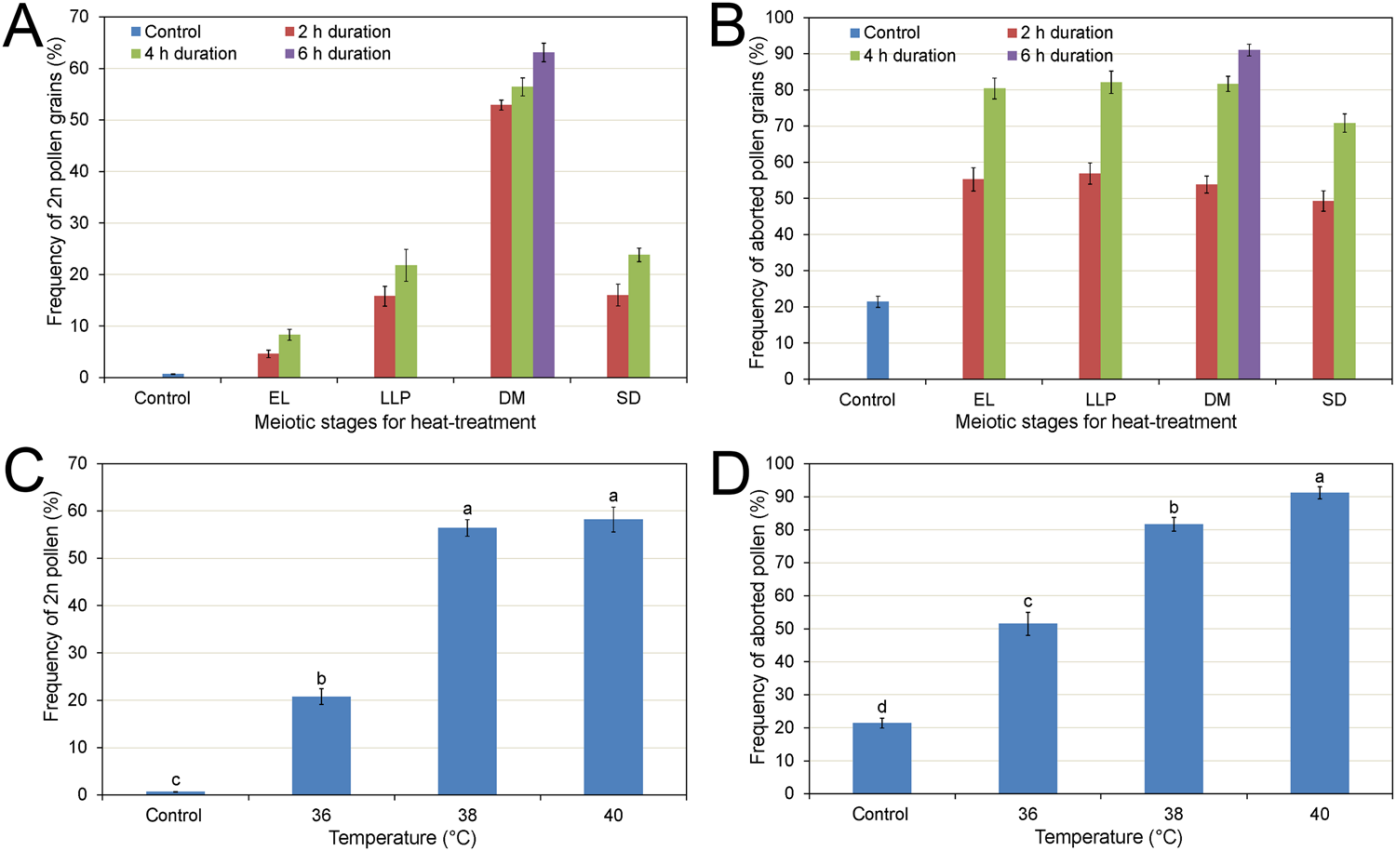
Histogram of the frequency of heat-induced 2*n* pollen grains and aborted grains. Heat treatments with 38 °C at various meiotic stages and treatment durations induced different frequencies of 2*n* pollen grains (**A**) and aborted grains (**B**). EL, early leptotene; LLP, late leptotene to pachytene; DM, diakinesis to metaphase I; SD, the second division. Different temperature treatments for 4 h at the diakinesis to metaphase I stage affected frequencies of 2*n* pollen (**C**) and aborted pollen (**D**). Values are mean ± SE of three replicates.

Although *P. pseudo-simonii* could spontaneously produce large pollen at a frequency of 0.66% (±0.09), high temperature treatments increased the proportion of large pollen markedly (Fig. 3A). Depending on the meiotic stage at the time of treatment, treatment at 38 °C could produce large pollen at a frequency of more than 50%. For treatments at the DM stage, proportions of large pollen were significantly higher than any other stage (*P* < 0.0001 both for 2- and 4-h durations), suggesting that the DM stage might be more suitable for high temperature-induced large pollen production. High temperature treatment at the second division (SD) stage also effectively induced large pollen at frequencies of 16.02% (±2.13) and 23.80% (±1.31) for the 2- and 4-h treatments, respectively. Although the frequency of large pollen production at the SD stage was significantly lower than that at the DM stage treatments (*P* = 0.0002 for 2-h and *P* < 0.0001 for 4-h durations), there were non-significant differences in the frequency of large pollen between the late leptotene to pachytene (LLP) stage and the SD stage for both the 2- and 4-h durations (*P* = 0.9457 and 0.5613, respectively).

The duration of treatment also affected the frequency of large pollen. Treatment for 6 h produced the highest percentage [63.09% (±1.81)] of large pollen in the DM stage treatments at 38 °C, which was significantly more efficient at large pollen induction than the 2- and 4-h treatments (*P* = 0.0061 and 0.0202, respectively). There was non-significant difference between the 2- and 4-h treatments (*P* = 0.1794) on the frequency of large pollen.

However, the heat stress affected the floral and pollen developments. The 6-h treatments in the DM stage restrained the amount of pollen. The buds treated at the early leptotene (EL) and LLP stages died and buds at the SD stage were not able to shed after treatment at 38 °C for 6 h. The 2- and 4-h treatments reduced the damage to pollen development and allowed for more pollen to be produced compared to the 6-h treatment, indicating that a long exposure to heat tended to terminate development of microsporocytes. In the collected pollen samples, the frequencies of the high temperature-induced aborted grains, ranged from 49.27% to 91.01% for the different durations and meiotic stages at 38 °C and they were all significantly higher than that of the control, which was 21.40% (±1.52) (*P* = 0.0001, Fig. 3B). In the 6-h treatment at the DM stage, the frequency of aborted grains reached 91.01% (±1.62). In the 2-h treatments, the difference of the meiotic stages on the production of aborted grains was not significant. However, the rate of aborted pollen in the 4-h treatments of the EL, LLP, and DM stages were all significantly higher than that of the SD stage (*P* = 0.022), although there was non-significant difference among the frequencies of aborted pollen for the three stages. This finding indicated that the meiotic stages of prophase I were less resistant than during the second division.

The effect of temperature on the production of large pollen and aborted grains was investigated using treatments of 36, 38, 40, and 42 °C for 4 h at the DM stage. In the 42 °C treatments, no pollen was collected due to failure in anther dehiscence. In the other treatments, the frequency of high temperature-induced large pollen increased gradually with the increase in temperature. The frequencies of large pollen were 20.78% (±1.65) at 36 °C, 56.41% (±1.74) at 38 °C, and 58.19% (±2.62) at 40 °C, respectively, which were all significantly higher than the control [0.66% (±0.09)] (*P* < 0.0001, Fig. 3C). Non-significant difference in the production of large pollen was found between the 38 and 40 °C treatments (*P* = 0.5198). The proportion of aborted pollen grains was 51.50% (±3.50) at 36 °C, 81.66% (±2.10) at 38 °C, and 91.21% (±1.86) at 40 °C, indicating an increased tendency for pollen to abort at high temperatures. The frequencies of aborted grains were significantly higher in all high-temperature treatments than in the control (*P* < 0.0001, Fig. 3D).

### High temperature-induced meiotic abnormalities

**Figure 4:**
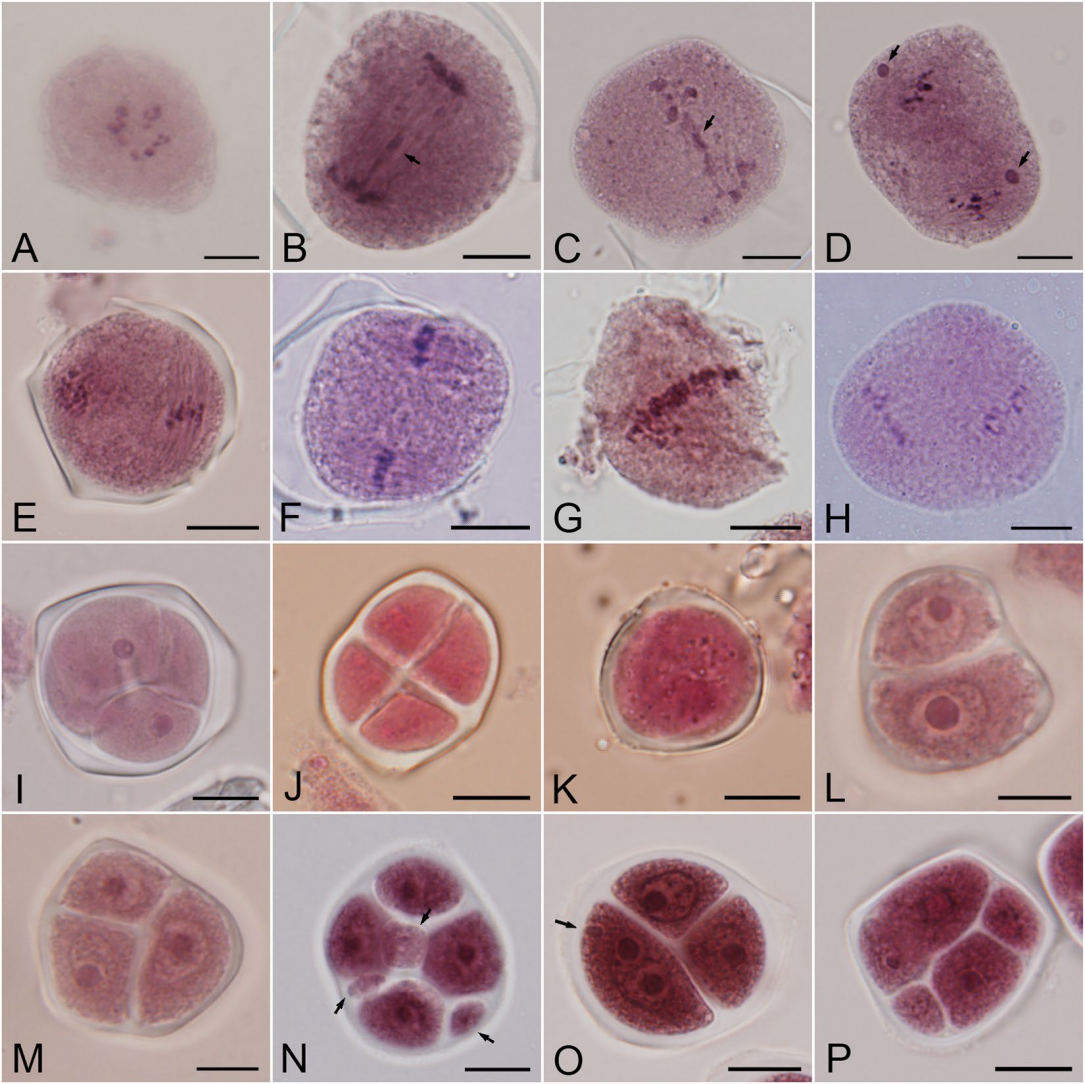
High temperature-induced abnormal meiotic chromosome behaviours, spindle disorientation and aberrant cytokinesis. (**A**) Irregular chromosome distribution in metaphase I. (**B**) Laggards (arrow) in anaphase I. (**C**) Chromosome stickiness (arrow) in telophase I. (**D**) Micronuclei (arrows) in metaphase II. (**E**) Normal perpendicular metaphase II spindles. (**F**) Spindles with parallel arrangement. (**G**) Fused spindles. (**H**) Tripolar spindles. (**I**) Normal tetrahedral tetrad. (**J**) Tetragonal tetrad. (**K**) Monad. (**L**) Dyad. (**M**) Triad. (**N**) Tetrad with three microcytes (arrows). (**O**) Triad with micronuclei (arrow) from the primary nuclei. (**P**) Polyad with unbalanced cytokinesis. Bars = 10 μm.

In the control samples, the microsporocytes underwent normal meiosis (see Wang *et al*.^37^). In the high temperature-treated samples, however, there were many abnormalities that occurred in meiosis. In metaphase I, the chromosomes spread irregularly in the cytoplasm (Fig. 4A) instead of localising on the sides of the equatorial plane, resulting in separation failure. Some chromosomes lagged in anaphase I and remained in the equatorial region (Fig. 4B), and chromosome stickiness was also observed in telophase I (Fig. 4C). Moreover, high temperature treatment also led to formation of micronuclei during the second division (Fig. 4D).

Perpendicular metaphase II spindles (Fig. 4E) were usually observed under normal conditions, but heat stress increased the frequency of spindle disorientation in the second meiotic division. For samples treated at 38 °C for 4 h during the DM stage, the frequency of parallel spindles (Fig. 4F) was 26.86% (±1.06), whereas, in the control samples the frequency of parallel spindles was 17.33% (±1.03). Additionally, two new types of particular spindle orientations, fused and tripolar spindles (Fig. 4G and H), were observed at a frequency of 32.80% (±1.57) and 23.96% (±0.90), respectively.

**Figure 5:**
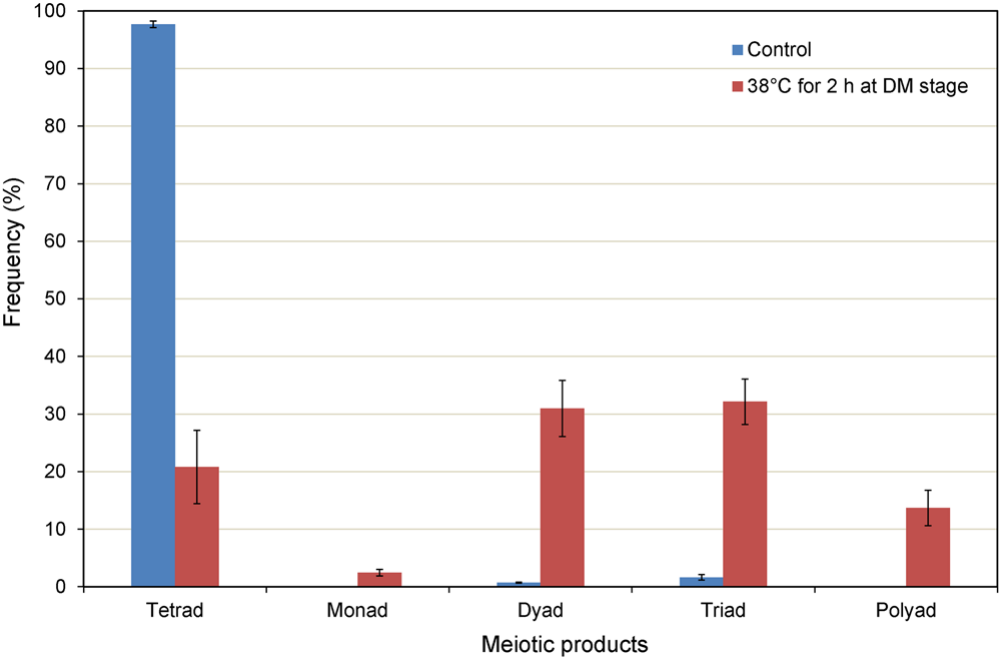
Production of meiotic products after treatment at 38 °C for 2 h during the diakinesis to metaphase I stage. DM, the diakinesis to metaphase I stage. Values are mean ± SE of three replicates.

Normal cytokinesis occurred in control samples, resulting in formation of tetrahedral and tetragonal arranged tetrads (Fig. 4I and J) depending on the spindle orientation. However, we found that the high temperature treatment tended to trigger aberrant cytokinesis, which produced a number of monads (2.42% ± 0.57), dyads (30.96% ± 4.85), triads (32.14% ± 3.96), and polyads (13.68% ± 3.10) in samples treated at 38 °C for 4 h during the DM stage (Figs 4K–P and 5). Several spores contained micronuclei that were not incorporated into primary nuclei, but rather remained in the cytoplasm with the primary nuclei (Fig. 4O). The size of spores from polyads was usually unbalanced (Fig. 4P), suggesting that the chromosomes might segregate asymmetrically and lead to production of aneuploid gametes.

### High temperature-induced defects of meiotic microtubular cytoskeletons

**Figure 6:**
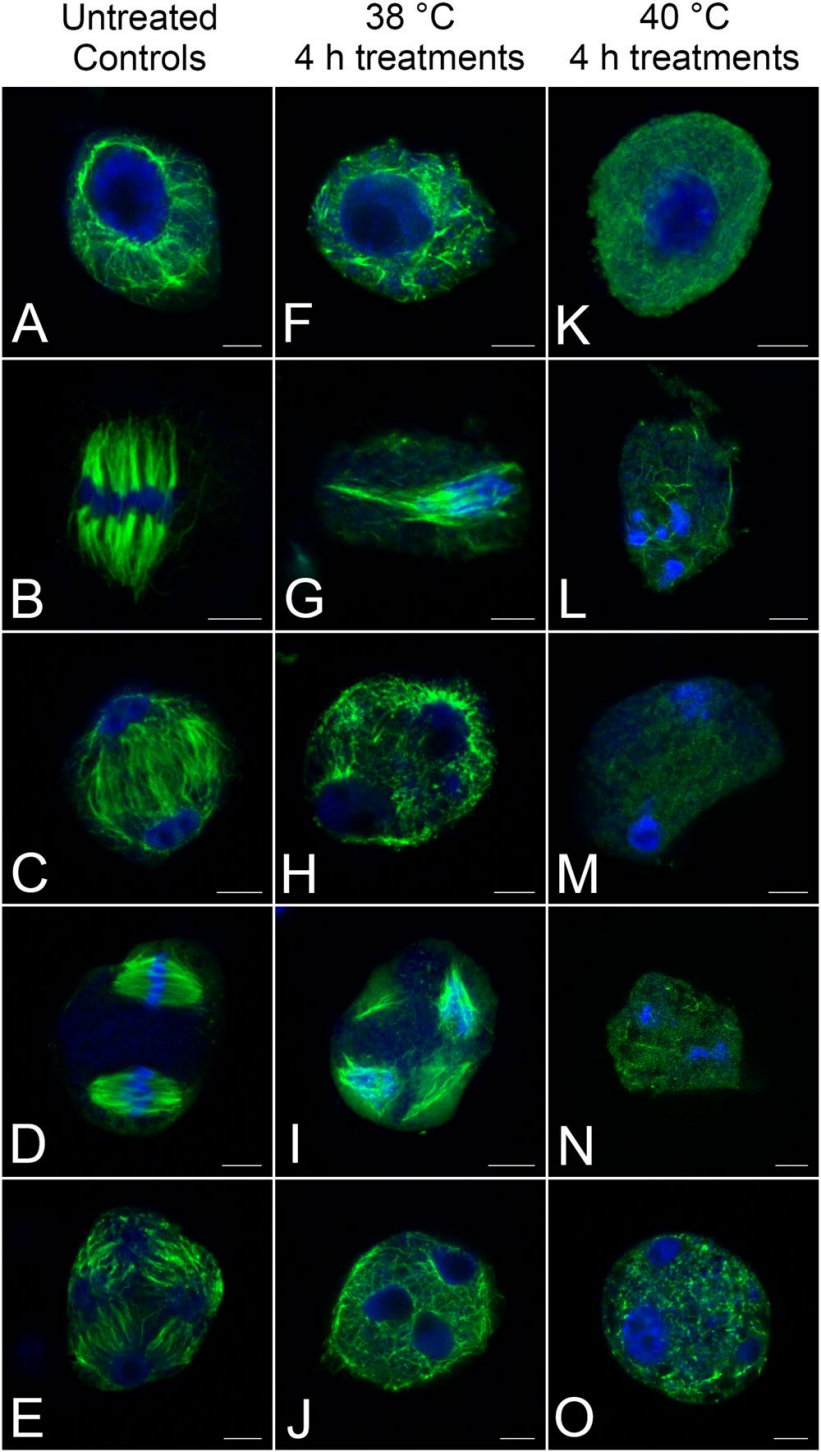
Defects in microtubule distribution and chromosome behaviours induced by high temperature treatments. (**A–E**) Microtubular distributions and chromosome behaviours under normal conditions. (**A**) The network of microtubules in prophase I. (**B**) Connections between chromosomes and microtubules in a metaphase I dipolar spindle. (**C**) Phragmoplast microtubules located between two daughter nuclei in telophase I. (**D**) Two spindles with parallel arrangement in metaphase II. (**E**) Nuclear-based RMSs and four separated daughter nuclei in telophase II. (**F–J**) Defects in microtubular arrangement and abnormal chromosome behaviours after treatment at 38 °C for 4 h. (**F**) Microtubules become fragments in prophase I. (**G**) Metaphase I spindle damage, resulting in unrestrained chromosomes in the cytoplasm. (**H**) High temperature treatment resulting in destroyed phragmoplast with a micronucleus. (**I**) Damaged spindles in metaphase II. (**J**) Failure of RMS formation due to heat stress. (**K–O**) Damages to the microtubular arrangement and chromosome behaviours after treatment at 40 °C for 4 h. (**K**) A cell in prophase I shows almost no microtubules. (**L**) A few microtubular fragments remaining in the cytoplasm of a cell in metaphase I. (**M**) Completely depolymerised microtubules in telophase I. (**N**) No visible spindles and just a few microtubular fragments in metaphase II, resulting in excessive condensation of chromosomes. (**O**) Depolymerised microtubules causing fusion of nuclei and formation of micronuclei in telophase II. Bars = 5 μm.

Under normal conditions, the microtubular cytoskeleton was distributed regularly in microsporocytes (Fig. 6A–E), which allowed for proper segregation of chromosomes in daughter cells. However, the high temperature treatment induced defects in the meiotic microtubular cytoskeleton. For example, the microtubules depolymerised and were not even present in the cytoplasm. The extent of the treatment affected the amount of microtubule destabilisation, and the plasma membrane even ruptured in some cells.

In the prophase of the first meiotic division, microtubules became fragments in cells treated at 38 °C for 4 h (Fig. 6F) and almost disappeared in cells treated at 40 °C for 4 h (Fig. 6K), whereas control cells arranged the microtubule network (Fig. 6A). In metaphase I, the spindle was damaged in cells treated at 38 °C for 4 h (Fig. 6G) and depolymerised in cells treated at 40 °C for 4 h (Fig. 6L). The damaged spindle resulted in chromosomes moving freely within the cytoplasm. In the control telophase I microsporocytes, phragmoplasts were observed in the equatorial region (Fig. 6C). In cells treated at 38 °C for 4 h, however, the phragmoplasts were destroyed (Fig. 6H) and in cells treated at 40 °C for 4 h the microtubules had completely depolymerised (Fig. 6M). The high temperature-treatment at 38 °C for 4 h damaged the polarity of spindles in cells at metaphase II (Fig. 6I), but control cells contained a regular set of spindles (Fig. 6D). After treatment at 40 °C for 4 h, the microtubules disappeared completely, resulting in excessive condensation of chromosomes (Fig. 6N). Under normal growth conditions, typical nuclear-based radial microtubule systems (RMSs) formed in telophase II (Fig. 6E). Primary phragmoplasts developed between sister nuclei and two narrow secondary phragmoplasts developed simultaneously between non-sister nuclei, which determined the position of cell plates. In cells treated at 38 °C for 4 h, however, the microtubules were not able to develop into RMSs and no phragmoplasts formed even though the microtubular network was visible (Fig. 6J). Cells treated at 40 °C for 4 h had fragmented microtubules, which caused fusion of nuclei and formation of micronuclei (Fig. 6O).

### Restoration of microtubular cytoskeletons after high temperature treatment supported completion of cytokinesis

**Figure 7:**
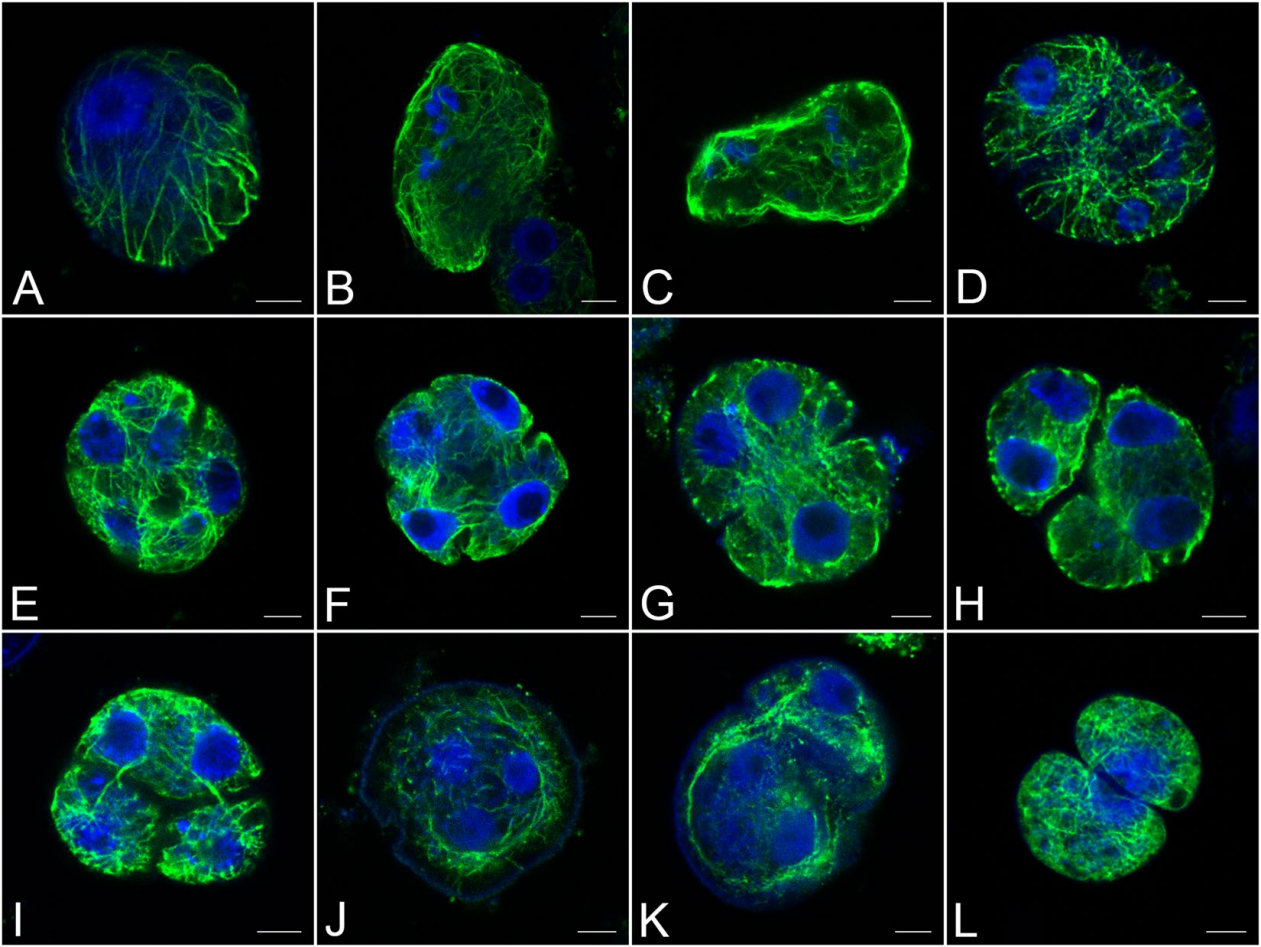
Restoration of microtubules after transferring heat-treated buds to normal conditions. (**A**) Several thick microtubular bundles reformed around the nucleus in prophase I after transferring to normal conditions for 18 h. (**B**,**C**) Repolymerised microtubular array instead of spindles in the cytoplasm of metaphase I (**B**) and metaphase II (**C**) cells after 12 h transferred. (**D**) Repolymerisation of microtubules in telophase II after 12 h transferred. (**E**) Formation of new microtubular networks and radial microtubule systems (RMSs) in a treated telophase II cell. (**F**) Cytoplasmic infurrowing between daughter nuclei. (**G–K**) Aberrant cytokinesis due to defects in the new RMSs between daughter nuclei. (**G**) A dyad with two unseparated nuclei in one microspore and one fused nucleus in the other microspore. (**H**) A dyad with one microcyte, showing two unseparated nuclei in each microspore. (**I**) A triad showing two nuclei in one microspore. (**J**) A monad resulted from failure in cytokinesis. (**K**) Unbalanced dyad (one nucleus in one microspore and three nuclei in the other microspore), showing a lack of RMSs between the unseparated nuclei. (**L**) An infurrowed cell with one shared fused nucleus. Bars = 5 μm.

Indirect immunofluorescence of microtubules showed that the microtubular cytoskeletons were able to recover in some heat-treated cells after transferring them to normal conditions. We found that the microtubules were likely to undergo repolymerisation close to the cytoplasmic membrane, where we observed a high intensity of microtubular fluorescence. In some heat-treated prophase I microsporocytes, thick microtubular bundles reformed around the nucleus several hours after transferring them to normal conditions for 18 h (Fig. 7A). In some heat-treated metaphase I and II cells, microtubular arrays repolymerised in the cytoplasm after 12 h transferred (Fig. 7B and C) instead of the microtubular spindle, as found in normal cells, which resulted in defects in chromosome segregation. The repolymerisation of microtubules also occurred at telophase II (Fig. 7D) after heat-treated cells were returned to normal conditions for 12 h and the microtubules developed new networks and RMSs (Fig. 7E). The repolymerised microtubules carried out cytokinesis accompanied by cytoplasmic infurrowing (Fig. 7F), which suggested that the restored microtubules were probably capable of completing cytokinesis. However, defects in new RMSs between daughter nuclei resulted in aberrant cytokinesis characterising by formation of monads, dyads, triads, and microcytes that contained micronuclei (Fig. 7G–J). Unbalanced dyads were formed by unequal cytokinesis due to a lack of RMSs between the unseparated nuclei (Fig. 7K). An infurrowed cell with one shared, fused nucleus (Fig. 7L) was also observed, which might be an explanation for the formation of conjoined pollen.

### High temperature-induced ultrastructural changes in tapetal cells

**Figure 8:**
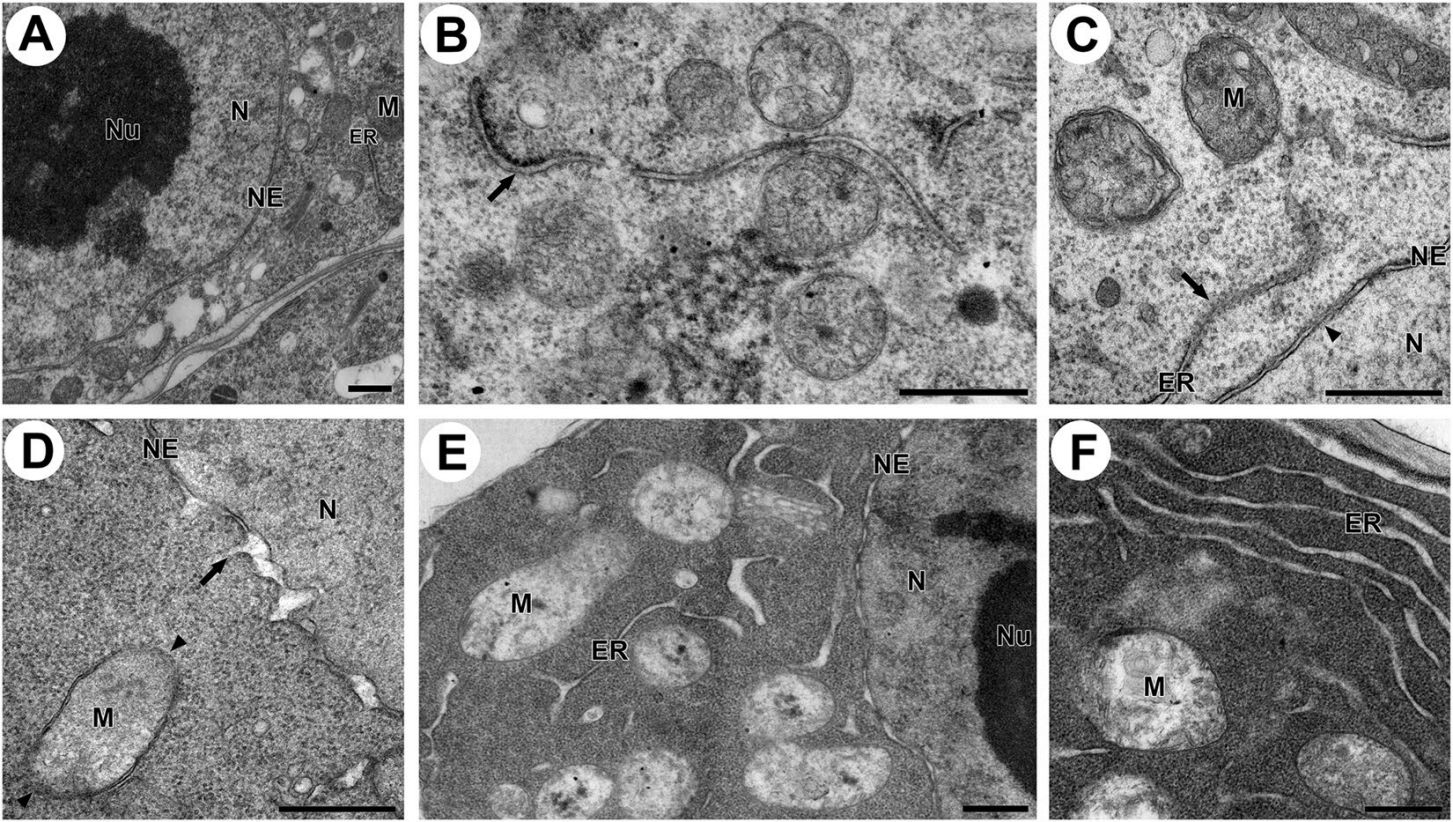
Transmission electron micrographs of tapetal cells. (**A**) Structure and distribution of organelles in a control cell. (**B**) Formation of smooth endoplasmic reticulum (arrow) lacking ribosomes under high temperature conditions. (**C**) Degradation of components of the ER (arrow) and NE (arrow head) by high temperature treatment. (**D**) Swollen and reptured NE lumen (arrow), and degradation of the outer membrane of mitochondria (arrow head) after high temperature treatments. (**E**) Swollen and ruptured NE and ER lumens after high temperature treatment. (**F**) Highly electron dense cytoplasm and swollen ERs after high temperature treatment. N, Nucleus; Nu, Nucleolus; NE, Nuclear envelope; M, Mitochondrion; ER, Endoplasmic reticulum. Bars = 0.5 μm.

The transmission electron microscopy (TEM) analyses demonstrated that heat stress induced negative structural changes in tapetal cells at the sub-cellular level. Under control conditions, the tapetal cells were regular, exhibiting proper distribution and structure of the organelles (Fig. 8A). Rough endoplasmic reticulum (RER) was arranged linearly in the cytoplasm and was covered with ribosomes. The nuclear envelope (NE) had an entire double-membrane structure. Mitochondria also had regular shape and inner structure. However, high temperature treatments induced structural changes in the organelles. With moderate high temperature treatment (38 °C for 2 h), smooth endoplasmic reticulum (SER), which lack ribosomes on the surface, was sometimes visible and the outer membrane of several mitochondria shrank, causing alterations in the shape of the mitochondria (Fig. 8B). Higher intensity heat treatments (38 °C for 4 h or 40 °C for 2 h) induced swelling of the mitochondrial saccules and degradation of parts of the endoplasmic reticulum (ER) and NE (Fig. 8C). Further increases in temperature resulted in degradation of the outer membrane of some mitochondria (Fig. 8D). The lumen of the ER and NE also swelled and ruptured (Fig. 8D and E). The cytoplasm of heat-treated cells became highly electron dense (Fig. 8F) compared to the control.

## Discussion

In this study, we successfully induced unreduced pollen of *P. pseudo-simonii* using high temperature treatments. It is well known that the ploidy level of pollen can be distinguished by analysing pollen size and shape^38–41^. In unreduced pollen producers of alfalfa, pollen diameters usually follow a bimodal distribution^42^. The histogram of pollen diameter distribution in this study indicated that unreduced pollen was successfully induced by high temperature treatments, although there still was haploid pollen in the pollen samples. Diploid eggs are also known to be induced by high temperatures, as a large number of triploids were produced though pollination with normal pollen in *Populus* ^34, 35^. These findings suggested that high temperature is an efficient environmental factor for both male and female unreduced gamete induction^6, 16^.

We found that the temperatures required for 2*n* pollen induction of *P. pseudo-simonii* were 38–40 °C in this study, which is same with white poplar^43^. However, in previous studies of high temperature-induction of 2*n* eggs in *Populus*, the most suitable treatments were 41–44 °C depending on whether the treatment occurred during megasporogenesis or embryo sac development^34, 35^, which was higher than the temperatures for 2*n* pollen induction. This suggested that female gametic development might be less sensitive to high temperature stress than microsporogenesis^44^.

In this study, the developmental stage from diakinesis to metaphase I was found to be the most suitable time period for high temperature-induction of 2*n* pollen in *Populus*, which is a more exact window than that reported in *Rosa* ^17^. The meiotic stage is the key factor for efficiency of unreduced gamete induction^45^. For colchicine-induced 2*n* pollen and megaspore production in *Populus*, the pachytene stage of meiosis was found to be the optimal period for treatment^46, 47^, which is slightly earlier than that for high temperature treatment, possibly because the conduction of heat inside the anthers was faster than the diffusion of the colchicine solution.

Unreduced pollen was also induced by high temperatures during the second meiotic division in this study, although the frequency of unreduced pollen was significantly lower than that in diakinesis to metaphase I stage, suggesting that meiotic inhibition during the second meiotic division can also produce 2*n* pollen. According to the known mechanisms of 2*n* gamete formation, the induced 2*n* pollen resulted from failed chromosome separation during the second meiotic division could be defined as the SDR type and the 2*n* pollen induced at prophase I could be the FDR type. In previous study, both FDR and SDR type 2*n* eggs were also induced in *Populus* ^35^. Dong *et al*.^48^ analysed the transmission of heterozygosity of FDR and SDR type 2*n* eggs in *Populus* and found that they transmitted 74.80 and 39.58% maternal heterozygosity, respectively. For the 2*n* pollen induced in this study, there might be a similar transmission rate of paternal heterozygosity. The heterozygosity of 2*n* gametes could contribute to increased genotypic diversity of polyploid progeny^16^.

With global climate changing caused by the greenhouse effect, response of plant reproduction to dramatic fluctuations of temperature has attracted increasing attention^49^. Our results showed that high temperature treatment resulted in numerous meiotic abnormalities, including chromosome stickiness, laggards, micronuclei, spindle disorientations in the second meiotic division and aberrant cytokinesis, which suggests that meiosis is vulnerable to high temperature^50, 51^. These abnormal meiotic chromosome behaviours likely led to high frequency of pollen abortion. Furthermore, malfunction of tapetum can also lead to pollen sterility, since it plays a major role in the nutrition of microspores during microsporogenesis^52^. In this study, high temperature treatment resulted in ultrastructural damage to tapetal cells, such as formation of SER, swelling and degradation of mitochondrial saccules and ER lumen, and rupture of ER and NE, as observed in *Phaseolus vulgaris* ^53^. These ultrastructural damages of endomembrane system blocked their function in the tapetum and also caused production of aborted pollen. Additionally, He *et al*.^54^ found that ultrastructural changes in pollen mother cells of autotetraploid rice were related with pollen sterility and low seed set. This suggested that ultrastructural changes in microsporocytes might occur after high temperature treatments.

In addition to gamete sterility, spindle disorientations are also relevant to the production of unreduced gametes^17, 22, 33^. In this study, parallel, fused and tripolar spindles were recorded with high frequencies after high temperature treatments. These disorientations of spindles likely resulted in the formation of dyads and triads in meiotic products. However, for spontaneous formation of 2*n* pollen in *Populus*, it is found that the occurrence of parallel spindles was not correlated with formation of dyad, because an organelle band was located in cytoplasm to prevent the coalescence of spindles^25, 55^.

Monads and polyads were also observed after high temperature treatments in this study. The monads could develop into pollen grains with polyploidy level. The polyads might develop aneuploid pollen grains. Henry *et al*.^56^ found that whole chromosome deletions and insertions were induced by gamma irradiation in *P. nigra*, resulting in aneuploid offspring, which suggested that the aneuploid gametes might be viable. Once a full-sib population, including diploids, polyploids, and aneuploids, is established by pollination with high temperature-induced pollen, further studies of chromosome engineering and the effects of chromosome-dosage in *Populus* will be valuable.

Movement of chromosomes and cytokinesis during meiosis are both related to changes in the microtubular cytoskeleton^24, 57^. Colchicine-induced microtubular gathering inhibited chromosome segregation in the cytoplasm of microsporocytes during prophase I of *P. alba*, which resulted in 2*n* pollen formation^58^. However, depolymerisation of the meiotic microtubular cytoskeleton was induced by high temperature treatment in this study, resulting in failure of chromosome segregation. After returning the heat-treated cells to normal conditions, new microtubules repolymerised and were capable of completing cytokinesis. Microtubule-organising centres (MTOCs) are considered to be responsible for initiation of cytoplasmic microtubules in eukaryotic cells^59^. We assume that MTOCs may still be undamaged in some treated cells and function to initiate polymerisation of new microtubules during the post-treatment developmental process. However, although new microtubules polymerised in microsporocytes after high temperature treatment, defects in the formation of new RMSs in telophase II still occurred, resulting in partial failure of nuclei separation and aberrant cytokinesis. Thus, microtubular depolymerisation and incomplete restoration may be an important cytological mechanism for induction of unreduced gametes by high temperature. These findings might be particularly relevant for enhancing our understanding of how polyploidisation is induced by temperature stress.

Polyploidy breeding is one of the most efficient approaches to improve the genus *Populus*. Although many *Populus* triploid cultivars that exhibit improved growth and pulpwood properties have been used in plantation^60–62^, there is no triploid variety in section Tacamahaca. In this study, high temperature treatment induced both FDR and SDR type unreduced pollen with high frequency in *P. pseudo-simonii*, a species of section Tacamahaca, which could transmit different heterozygosity to allotriploid progeny. This is important for further variety selection in section Tacamahaca. However, maternal selection is necessary to produce triploid varieties with both heterosis and ploidy vigour.

## Materials and Methods

### Plant materials

An indigenous *Populus* species of China, *Populus pseudo-simonii* Kitag., was used to induce 2*n* pollen using high temperatures. Floral branches of *P. pseudo-simonii* were collected from an individual in a plantation in Tongliao City (Inner Mongolia Autonomous Region, P. R. China). After the branches were transported to Beijing Forestry University, they were water-cultured in a greenhouse (10–20 °C) for later use.

### High temperature treatments

As previously described by Wang *et al*.^37^, the floral morphology and the colour of anthers were used for preliminary determination of pollen development. The meiotic stage of microsporocytes was observed using the 1% acetocarmine squash method. To investigate the effects of the meiotic stage and the duration of treatments on polyploidisation, branches in the early leptotene (EL), late leptotene to pachytene (LLP), diakinesis to metaphase I (DM), and the second division (SD) stages of meiosis were treated at 38 °C for 2, 4, and 6 h. Branches in the DM stages of meiosis were treated at temperatures of 36, 38, 40, and 42 °C to analyse the effects of temperature on 2*n* pollen production. High temperature treatments were conducted in a phytotron chamber (Hadonglian, Harbin, China) with 60% relative humidity, a photosynthetic photon flux density (PPFD) of 200 μE m^−2^s^−1^, and a 16-h photoperiod. All treatments were conducted for three replications. Following treatment, the branches were transferred back to normal growth conditions in the greenhouse and meiotic development of the male flower buds was analysed.

### Cytological observation of microsporocytes

Before cytological observation of microsporogenesis, the control buds and heat-treated buds were fixed in ethanol-acetic acid fixative solution (3:1 v/v) at 4 °C for 24 h and stored in 70% ethanol for later analysis.

Microsporocytes or microspores were pressed out of anthers and squashed in 1% acetocarmine on a microscope slide. Then, the preparation was heated slightly over a flame to increase chromosome stainability. All preparations were observed using a microscope (Olympus BX51) and photos were taken using an attached CCD video camera (Olympus DP70). Abnormal meiotic chromosome behaviours and disorientated spindles were recorded based on at least 300 cells for each stage. Variations in the type and shape of sporads were analysed based on at least 700 cells for each sample.

### Identification of abnormal pollen grains

Pollen samples were collected from the control and heat-treated catkins and were observed microscopically after acetocarmine staining. Based on the findings of Mashkina *et al*.^32^, the spherical pollen grains with a diameter exceeding 37 μm were considered to be diploid pollen grains. The empty and shrunken pollen grains were considered to be aborted. Because the aborted pollen will be not valuable for sexual polyploidisation, they were excluded for calculating frequency of 2*n* pollen. The frequency of 2*n* pollen was calculated as the ratio of the number of large spherical pollen grains to the total number of spherical grains multiplied by 100. The frequency of aborted pollen was calculated as the ratio of the number of the empty and shrunken pollen grains to the total number of grains multiplied by 100. Some pollen grains with more than one grain conjoined together were photographed. The diameters of stained spherical pollen grains from normal untreated catkins and catkins treated at 38 °C for 2 h during the DM stage were measured using an ocular micrometer to analyse the response of pollen size to high temperature treatment. The frequency of large pollen grains was calculated and excluded the empty and shrunken pollen grains. For each sample, no less than 1,000 grains were analysed.

Mature pollen samples were stained to observe the vegetative and generative nuclei as described by Wang *et al*.^37^. Briefly, the pollen samples were hydrated for 1 h and fixed with 4% paraformaldehyde for 45 min. After extraction with 1% Triton X-100 for 30 min, samples were rinsed in distilled water and then stained with 4′,6-diamidino-2-phenylindole (DAPI). Microscopic observations were obtained using the Olympus BX51.

### Immunolocalisation of microtubular cytoskeletons

An indirect immunofluorescence test was used to analyse changes in the microtubular cytoskeleton as described by Wang *et al*.^26^. Briefly, anthers were fixed with 4% paraformaldehyde in a PEM buffer (50 mM Pipes, 5 mM EGTA, 2 mM MgSO_4_, pH 6.9) for 45 min. After being rinsed three times, the anthers were dipped in 10% dimethylsulfoxide (DMSO) for 15 min and then extracted with 1% Triton X-100 for 30 min. Subsequently, they were rinsed in the PEM buffer and then rinsed three times in a PBS buffer (137 mM NaCl, 2.7 mM KCl, 7 mM Na_2_HPO_4_, 1.5 mM KH_2_PO_4_, pH 7.3). Microsporocytes were squeezed out from the anthers on a slide coated with 0.1% poly-L-lysine (Sigma-Aldrich). The cells were incubated successively with a monoclonal anti-α-tubulin antibody (Sigma-Aldrich) diluted 1:100 and an FITC-conjugated anti-mouse IgG (Sigma-Aldrich) diluted 1:50 with PBS buffer for 2 h at 37 °C in a dark chamber. After a final wash in PBS buffer, the samples were mounted using Vectashield mounting medium with DAPI (Vector Laboratories). The preparations were observed and photographed with a Leica TCS-SP5 Confocal Laser Scanning Microscope.

### Transmission electron microscopy (TEM)

For observation of subcellular structures, anthers treated at various temperatures at diakinesis to metaphase I stage were fixed with 3% glutaraldehyde in 0.1 M phosphate buffer (pH 7.2) for 24 h at room temperature. After washing the samples with 0.1 M phosphate buffer (pH 7.2) five times, they were post-fixed with 1% osmium tetraoxide in 0.1 M phosphate buffer for 10 h at 4 °C. They were dehydrated in a graded alcohol series and embedded in Spurr’s resin. Ultrathin sections (60 nm) were prepared with diamond knives on a Leica EM UC6 microtome. The sections on grids were stained with 2% uranyl acetate for 20 min followed by a lead citrate staining solution for 5 min at room temperature. The sections were observed using a Hitachi H-7500 transmission electron microscope.

### Statistical analysis

Statistical analysis was carried out using SPSS software (Version 18.0). The results presented in this paper are mean ± SE. Before ANOVA analysis, the percentage data were transformed by the arcsin of the square root of p/100. When treatments were significantly different, a least significant difference (LSD) multiple comparison test was used for pairwise comparison. Because the data of pollen diameters failed the Shapiro test for normalcy (shapiro.test) and Bartlett test for homogeneity of variances (bartlett.test), the non-parametric Wilcoxon rank-test (wilcox.test) was performed in the R statistical environment^63^ to indicate the statistical difference of pollen size between control and treated groups.

### Data Availability

All data generated during the current study are available from the corresponding author on reasonable request.
